# Antimicrobial resistance of *Pseudomonas aeruginosa*: navigating clinical impacts, current resistance trends, and innovations in breaking therapies

**DOI:** 10.3389/fmicb.2024.1374466

**Published:** 2024-04-05

**Authors:** Ahmed Elfadadny, Rokaia F. Ragab, Maha AlHarbi, Farhad Badshah, Eliana Ibáñez-Arancibia, Ahmed Farag, Amin Omar Hendawy, Patricio R. De los Ríos-Escalante, Mohamed Aboubakr, Shadi A. Zakai, Wedad M. Nageeb

**Affiliations:** ^1^Laboratory of Internal Medicine, Cooperative Division of Veterinary Sciences, Tokyo University of Agriculture and Technology, Fuchu, Japan; ^2^Department of Internal Medicine, Faculty of Veterinary Medicine, Damanhour University, Damanhour, Egypt; ^3^Department of Biochemistry, Faculty of Veterinary Medicine, Damanhour University, Damanhour, Egypt; ^4^Department of Biology, College of Science, Princess Nourah Bint Abdulrahman University, Riyadh, Saudi Arabia; ^5^State Key Laboratory of Animal Biotech Breeding, Institute of Animal Science, Chinese Academy of Agricultural Science, Beijing, China; ^6^PhD Program in Sciences Mentioning Applied Molecular and Cell Biology, La Frontera University, Temuco, Chile; ^7^Laboratory of Engineering, Biotechnology and Applied Biochemistry – LIBBA, Department of Chemical Engineering, Faculty of Engineering and Science, La Frontera University, Temuco, Chile; ^8^Department of Biological and Chemical Sciences, Faculty of Natural Resources, Catholic University of Temuco, Temuco, Chile; ^9^Department of Surgery, Anesthesiology, and Radiology, Faculty of Veterinary Medicine, Zagazig University, Zagazig, Egypt; ^10^Department of Animal and Poultry Production, Faculty of Agriculture, Damanhour University, Damanhour, Egypt; ^11^Nucleus of Environmental Sciences, Faculty of Natural Resources, Catholic University of Temuco, Temuco, Chile; ^12^Department of Pharmacology, Faculty of Veterinary Medicine, Benha University, Moshtohor, Toukh, Qaliobiya, Egypt; ^13^Department of Clinical Microbiology and Immunology, Faculty of Medicine, King Abdulaziz University, Jeddah, Saudi Arabia; ^14^Department of Medical Microbiology and Immunology, Faculty of Medicine, Suez Canal University, Ismailia, Egypt

**Keywords:** *Pseudomonas aeruginosa*, resistance, pathogenicity, virulence, therapy

## Abstract

*Pseudomonas aeruginosa*, a Gram-negative bacterium, is recognized for its adaptability and opportunistic nature. It poses a substantial challenge in clinical settings due to its complicated antibiotic resistance mechanisms, biofilm formation, and capacity for persistent infections in both animal and human hosts. Recent studies revealed a potential zoonotic transmission of *P. aeruginosa* between animals, the environment, and human populations which highlights awareness of this microbe. Implementation of the One Health approach, which underscores the connection between human, animal, and environmental health, we aim to offer a comprehensive perspective on the current landscape of *P. aeruginosa* management. This review presents innovative strategies designed to counteract *P. aeruginosa* infections. Traditional antibiotics, while effective in many cases, are increasingly compromised by the development of multidrug-resistant strains. Non-antibiotic avenues, such as quorum sensing inhibition, phage therapy, and nanoparticle-based treatments, are emerging as promising alternatives. However, their clinical application encounters obstacles like cost, side effects, and safety concerns. Effectively addressing *P. aeruginosa* infections necessitates persistent research efforts, advancements in clinical development, and a comprehension of host-pathogen interactions to deal with this resilient pathogen.

## 1 Introduction

*Pseudomonas aeruginosa*, a Gram-negative bacterium of paramount significance, resides predominantly within healthcare settings, where it assumes a pivotal role as a causative agent of nosocomial infections (Moradali et al., 2017; Reynolds and Kollef, 2021). Simultaneously, it exerts its pathogenicity through opportunistic infections in diverse hosts, including humans, animals, and plants. The challenge of treatment of *P. aeruginosa* is always due to its ability to survive under a spectrum of environmental conditions and its resistance to available antimicrobial agents (Laborda et al., 2021). A distinctive hallmark of *P. aeruginosa* is its propensity for biofilm formation, an intricate process wherein bacterial cells aggregate within a self-produced extracellular matrix. This biofilm formation, encompassing a complex architectural arrangement, renders the bacterium impervious to conventional antibiotic regimens and contributes significantly to its clinical impact (Sharma et al., 2023). In a paradoxical twist, these biofilm structures have been observed to enhance the bacterium’s vulnerability when exposed to certain stressors such as antimicrobial agents, ultraviolet (UV) light, and variations in salinity (Roy et al., 2018; Sharma et al., 2019).

The prevalent presence of *P. aeruginosa* in nosocomial settings is not limited to its role as a colonizer of biotic and abiotic surfaces but extends to its capacity to form biofilms on medical devices, implants, and instrumentation, thereby increasing the risk of infections in hospitalized patients (Muhammad et al., 2020). Moreover, its ability to develop multidrug resistance (MDR) is a compelling facet of concern, as it withstands a broad spectrum of antibiotic classes including aminoglycosides (amikacin, gentamicin, and tobramycin), fluoroquinolones (ciprofloxacin, ofloxacin, and norfloxacin), carbapenems, and tetracyclines (Avakh et al., 2023).

Beyond its clinical impact, *P. aeruginosa* extends its dominion into the realm of environmental microbiology. A ubiquitous environmental bacterium, it traverses various ecological niches with remarkable adaptability, aided by a genome characterized by plasticity and high nutritional versatility (Grosso-Becerra et al., 2014). Within this genetic diversity lies an extensive range of intrinsic and acquired resistance mechanisms that collectively contribute to the bacterium’s impressive ability to circumvent antimicrobial agents. Understanding the complexity of resistance mechanisms and their interplay within the *P. aeruginosa* population is paramount for the development of targeted therapeutic interventions, particularly in the context of MDR strains. Our exploration into the world of *P. aeruginosa* will illuminate the adaptive strategies that cause its clinical and environmental persistence (Langendonk et al., 2021). In this pursuit of knowledge, our mission is to organize a comprehensive summary of the current epidemiological situation related to *Pseudomonas aeruginosa*, investigate the widespread issue of antimicrobial resistance and its underlying mechanisms, and establish a pathway for the discovery of alternative antimicrobial agents targeting this bacterium.

## 2 Clinical infections of *Pseudomonas aeruginosa*

**Figure 1:**
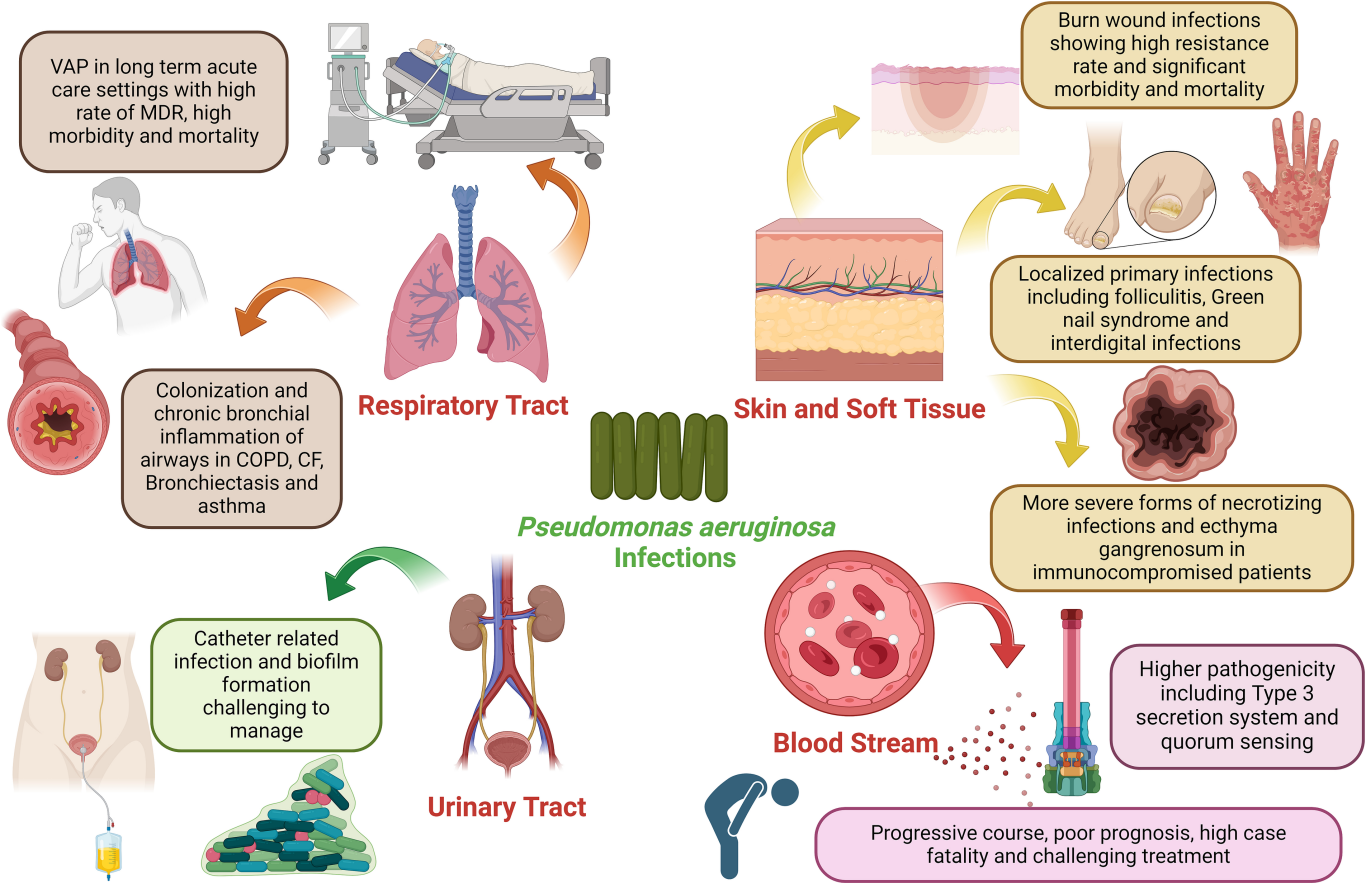
Clinical impact of *P. aeruginosa* infection in various organs of the human body, the figure was created with biorender.

*P. aeruginosa*, initially identified in 1882 through Gessard’s work involving green pus, exhibits a versatile lifestyle that enables it to contribute to frequent infections in humans (Lyczak et al., 2000). While it can be part of the normal intestinal flora, it does not readily adhere to healthy intact epithelium and typically does not cause infections in individuals in good health. However, it becomes an opportunistic pathogen in immunocompromised patients. Due to its adaptability and robust survivability, it can persist on dry, inanimate surfaces within hospital environments for a long period (Muggeo et al., 2023). It often contaminates healthcare equipment and various surfaces such as monitors, ventilator controls, bed rails, and respiratory devices. *P. aeruginosa* is implicated in a spectrum of hospital-acquired infections, spanning from ventilator-associated pneumonia (VAP) to bloodstream infections (Figure 1). It stands as the fourth most frequently isolated nosocomial pathogen, contributing to 10% of all hospital-acquired infections (Caffrey et al., 2022). A recent analysis of 8,247 inpatients with nosocomial infections across various tertiary care hospitals in Greece identified 746 patients with active healthcare-associated infections. *P. aeruginosa* emerged as the second most frequently isolated bacterium, accounting for 16% of cases, and was responsible for 49.4% of resistance against the carbapenem group of antibiotics (Kritsotakis et al., 2017). Notably, *P. aeruginosa* has been linked to higher mortality rates in bloodstream infections compared to infections caused by other Gram-positive and Gram-negative organisms.

### 2.1 *Pseudomonas aeruginosa* and respiratory infections

*Pseudomonas aeruginosa* is a prominent pathogen in healthcare settings, particularly among immunocompromised patients. It poses a significant threat as it can be transmitted through medical equipment and cross-contamination among patients (Wood et al., 2023). Ventilator-associated pneumonia (VAP) is a serious complication in patients kept on mechanical ventilation for more than 48 h in which *P. aeruginosa* is responsible for a high proportion of these infections in hospitalized patients. In the context of VAP, *P. aeruginosa’s* involvement is associated with higher case fatality rates than other bacterial causes. Tracheobronchial colonization is a critical factor in VAP, with *Staphylococcus aureus*, *P. aeruginosa*, and *Enterobacteriaceae* being the primary pathogens. High quantities of *P. aeruginosa* in ventilated patients’ tracheas correlate with an increased risk of death. The bacterium’s ability to resist conventional antibiotics, often due to complex drug-resistance genes present in plasmids or integrated into the bacterial genome, poses a significant challenge (Pachori et al., 2019). Studies have revealed that Pseudomonas spp. account for about 3–5% of nosocomial pneumonia cases, with *P. aeruginosa* being a prominent strain in ventilator-associated cases (Leroy et al., 2005). Nosocomial pneumonia secondary to *P. aeruginosa* has worse clinical outcomes when compared to other typical organisms. The prevalence of MDR *P. aeruginosa* VAP is reported to be generally high (33%) (Li et al., 2024). The crude mortality rate for *P. aeruginosa* VAP has been estimated at 42.1–87% even in patients receiving appropriate antimicrobial therapy (Foucrier et al., 2023). In addition to its impact on VAP, *P. aeruginosa* is among the top microorganisms responsible for healthcare respiratory infections. Infections caused by MDR-*P. aeruginosa* strains significantly increase the risk of mortality. Risk factors for VAP with *P. aeruginosa* include prior antibiotic exposure, airway colonization, and mechanical ventilation exceeding five days. On the other hand, *P. aeruginosa* is capable of chronic colonization, persistence, and infection of lower respiratory tracts in patients with chronic inflammatory airway diseases including asthma, chronic obstructive pulmonary disease (COPD), cystic fibrosis (*CF*), and bronchiectasis (Garcia-Clemente et al., 2020). Patients with chronic diseases like obstructive pulmonary disease and other respiratory conditions are also susceptible to the selection of hypermutator *P. aeruginosa* strains in chronic respiratory tract infections by *P. aeruginosa* facilitated by the frequent use of antimicrobials in these cases. Additionally, what makes *P. aeruginosa* particularly challenging is its ability to resist treatment, especially in the context of cystic fibrosis *(CF)* (Abdulwahab et al., 2017). In this hereditary disease, *P. aeruginosa* becomes a predominant pathogen in the lungs of affected individuals. Despite efforts to eradicate it, *P. aeruginosa* often persists, driving disease progression. It’s critical to understand how *P. aeruginosa* adapts and survives within the hyperinflammatory environment of the *CF* lung to develop effective treatments. Chronic bronchial infections in persons with *CF*, bronchiectasis, and COPD have been linked to more severe disease and poor prognosis. Bacterial colonization by *P. aeruginosa* has also been documented in people with chronic severe long-standing asthma with frequent exacerbations contributing to unfavorable clinical outcomes (Zhang et al., 2012).

*P. aeruginosa* presents multiple virulent factors and adapts through genotypic and phenotypic alterations to evade antibiotics and the immune system. The chronic infection environment within the *CF* lung subjects *P. aeruginosa* to significant stressors, including competition for resources, anaerobic conditions, high antibiotic concentrations, and immune responses like neutrophil attacks (Jurado-Martín et al., 2021). In response, *P. aeruginosa* undergoes microevolution, acquiring spontaneous mutations that lead to the selection of variants better suited for long-term colonization. These variants exhibit reduced virulence but greater resistance to antibiotics and host immunity. This adaptation includes the downregulation of virulence factors, increased biofilm formation, and upregulation of exopolysaccharides (Sousa and Pereira, 2014). These changes help *P. aeruginosa* avoid immune recognition, phagocytosis, and proinflammatory responses, promoting its persistence in the host. Moreover, *P. aeruginosa* forms biofilms within the *CF* lung, which significantly enhances its resistance to antibiotics and immune effectors. Biofilm communities appear as microcolonies or aggregates closely associated with the airway epithelium. They can evade the complement system, immobilize neutrophils, and resist the actions of neutrophil extracellular traps, further enabling *P. aeruginosa* to thrive within the *CF* lung (Maurice et al., 2018).

### 2.2 *Pseudomonas aeruginosa* and skin infections

*Pseudomonas aeruginosa* is a formidable pathogen with increasing clinical significance, particularly concerning skin and soft tissue infections. Its impact becomes far more pronounced in hospitalized patients due to its high resistance to a wide array of commonly available anti-pseudomonal agents (Wang et al., 2019). *P. aeruginosa* plays a critical role in burn wound infections, accounting for approximately one-third of all burn-related infections. These infections are notorious for their resistance to treatment, despite the availability of newer antibiotics with broad-spectrum activity (Gonzalez et al., 2016). Burned patients are particularly vulnerable, and infections caused by *P. aeruginosa* can lead to severe morbidity and, in some cases, mortality. The resistant nature of these infections not only prolongs hospital stays but also escalates treatment costs, representing a significant burden on healthcare systems. In skin infections, *P. aeruginosa* can cause a range of infections, from mild to severe, with unique clinical presentations. These infections typically affect otherwise healthy individuals and may sometimes resolve spontaneously without specific antibacterial therapy. Skin manifestations associated with *P. aeruginosa* infections may range from less severe localized forms of primary skin infections including Green Nail Syndrome, Interdigital Infections, and hot tub Folliculitis to more severe forms occurring in immunocompromised patients such as Ecthyma Gangrenosum and subcutaneous nodules usually occurring with bloodstream infections and also necrotizing skin and soft tissue infections occurring in diabetic, alcoholic and immunocompromised patients (Spernovasilis et al., 2021). *P. aeruginosa* plays an important role as a pathogen that extends beyond human infections; it also poses a significant threat to animals, including pets and livestock, with skin infections being a notable concern (Singh et al., 2005). In animals, *P. aeruginosa* can cause a range of dermatological conditions, including wound infections, hot spots, and otitis infections (de Sousa et al., 2023; Elfadadny et al., 2023b). These infections can lead to discomfort, pain, and, in severe cases, systemic illness. Veterinarians often encounter challenges in treating *P. aeruginosa* infections due to their ability to develop resistance to antibiotics, mirroring the issues seen in human medicine (Nielsen et al., 2022). The increasing trends of antibiotic resistance observed in this bacterium severely limit the choice of effective antimicrobial agents. Studies have demonstrated rising resistance rates of *P. aeruginosa* to numerous antibiotics, including those commonly used to combat skin and soft tissue infections.

### 2.3 *Pseudomonas aeruginosa* and ear infections

*Pseudomonas aeruginosa* can also invade the ears of both humans and animals. In humans, *P. aeruginosa* ear infections can lead to a painful condition known as swimmer’s ear or otitis externa. This infection often occurs after exposure to contaminated water, such as in swimming pools or hot tubs (Mittal et al., 2016). *P. aeruginosa* live in these moist environments, causing inflammation, itching, discharge, and sometimes even hearing difficulties or complicated to otitis media and malignant external otitis if left untreated. Treatment typically involves antibiotic ear drops, but the bacterium’s ability to develop resistance can complicate the therapeutic process (Sun et al., 2021). Malignant otitis externa is a serious invasive life-threatening condition that affects the external ear and skull base and can be complicated by mastoiditis and cranial nerve palsy if left untreated. It classically occurs in elderly, immunocompromised, and diabetic patients (Wu et al., 2011). Similarly, in animals, particularly dogs, cats, and other domestic pets, *P. aeruginosa* ear infections are a concerning issue. These infections can stem from various factors, including allergies, foreign objects in the ear canal, or underlying health conditions. *P. aeruginosa’s* adaptability and antibiotic resistance underscores the importance of prompt diagnosis and appropriate treatment to prevent further complications in both human and animal ear infections.

### 2.4 *Pseudomonas aeruginosa* and urinary tract infections

*Pseudomonas aeruginosa* has been increasingly implicated in UTIs. These infections represent a significant clinical concern, particularly in healthcare environments where indwelling urinary catheters are prevalent (Newman et al., 2022). Catheter-associated UTIs by *P. aeruginosa* are of substantial clinical consequence due to their association with pyelonephritis, prolonged morbidity, and mortality. *P. aeruginosa* contributes to approximately 10% of catheter-associated UTIs (CAUTI) and up to 16% of UTIs in ICU patients, with an alarming increase in nosocomial-acquired infections (Rosenthal et al., 2016; Farzin et al., 2023). The bacterium’s ability to exploit catheters as a tool for host entry underscores its clinical significance in UTIs. The insertion of urinary catheters can disturb mucosal epithelial layers, thereby promoting bacterial colonization and subsequent infection. Consequently, *P. aeruginosa* emerges as a dominant uropathogen in this clinical context. *P. aeruginosa* encompasses cell-associated factors such as alginate, lipopolysaccharides (LPS), flagella, and pili, alongside secretory virulence factors like proteases, elastases, toxins, and hemolysins (Mekonnen et al., 2022). Biofilm formation represents a hallmark of *P. aeruginosa’s* strategy to establish and persist in UTIs. The bacterium’s innate tendency to adhere to catheter surfaces leads to robust biofilm development. These biofilms present exceptional resistance against antimicrobial agents and host immune defenses. The ability of biofilms to promote persistent and recurrent infections is a pressing clinical concern in UTIs which renders *P. aeruginosa* infections challenging to manage (Mekonnen et al., 2022). Additionally, the ability of *P. aeruginosa* to invade the bladder epithelium has been recently demonstrated. Extensive evidence exists about the ability of *P. aeruginosa* to preferentially bind to, invade, and injure wounded epithelium. This ability to invade vulnerable epithelial cells has been recently proven to correlate well with the high prevalence of *P. aeruginosa* UTI in hospitalized elderly populations (Newman et al., 2022).

### 2.5 *Pseudomonas aeruginosa* and blood infections

Bloodstream infections (BSIs) caused by *P. aeruginosa* are emerging as a significant concern in clinical settings. *P. aeruginosa* is responsible for a significant number of bacteremia, ranking third, after *Escherichia coli* and *Klebsiella* species, among Gram-negative bacteria isolated during nosocomial bloodstream infections, and seventh among all pathogens (Holmes et al., 2021). Its significance arises from its place in case of fatality among all pathogens. Bacteremia caused by *P. aeruginosa* carries a poor prognosis, rapidly progressive course, and high mortality rate (Murray et al., 2022). Overall mortality associated with *P. aeruginosa* BSIs was recently reported at 26.8% (Shi et al., 2019). The incidence of *P. aeruginosa*-induced BSI is on the rise, contributing to elevated rates of morbidity and mortality among affected individuals. Understanding the sources and risk factors associated with these infections is crucial for effective management and prevention. Various studies have attempted to identify the origins of BSI associated with *P. aeruginosa*. Among these reports, respiratory tract infections and central venous catheters stand out as the most frequent sources. Several risk factors have been linked to *P. aeruginosa* BSIs. Patients who are immunocompromised, particularly those in intensive care units (ICUs), face an increased susceptibility to such infections (Bhardwaj et al., 2021). Furthermore, individuals with underlying conditions like lung cancer are at an increased risk. Previous antimicrobial therapy has also been identified as a risk factor, emphasizing the need for judicious and appropriate use of antibiotics. The severity of infection and pathogenesis of bloodstream infections have been linked to producing type III secretion-dependent exo-products and quorum-sensing dependent virulence (van Delden, 2007). During the COVID-19 pandemic, the overall incidence of BSIs including those caused by *P. aeruginosa* increased. *P. aeruginosa* BSIs overall increased among COVID-19 patients due to multiple factors including prolonged hospitalization, high-dose systemic corticosteroids treatment for acute respiratory distress in the ICU, and the administration of immune-modulator treatment such as tocilizumab or baricitinib (Bongiovanni and Barda, 2023).

## 3 Pathogenicity and antimicrobial resistance of *Pseudomonas aeruginosa*

**Figure 2:**
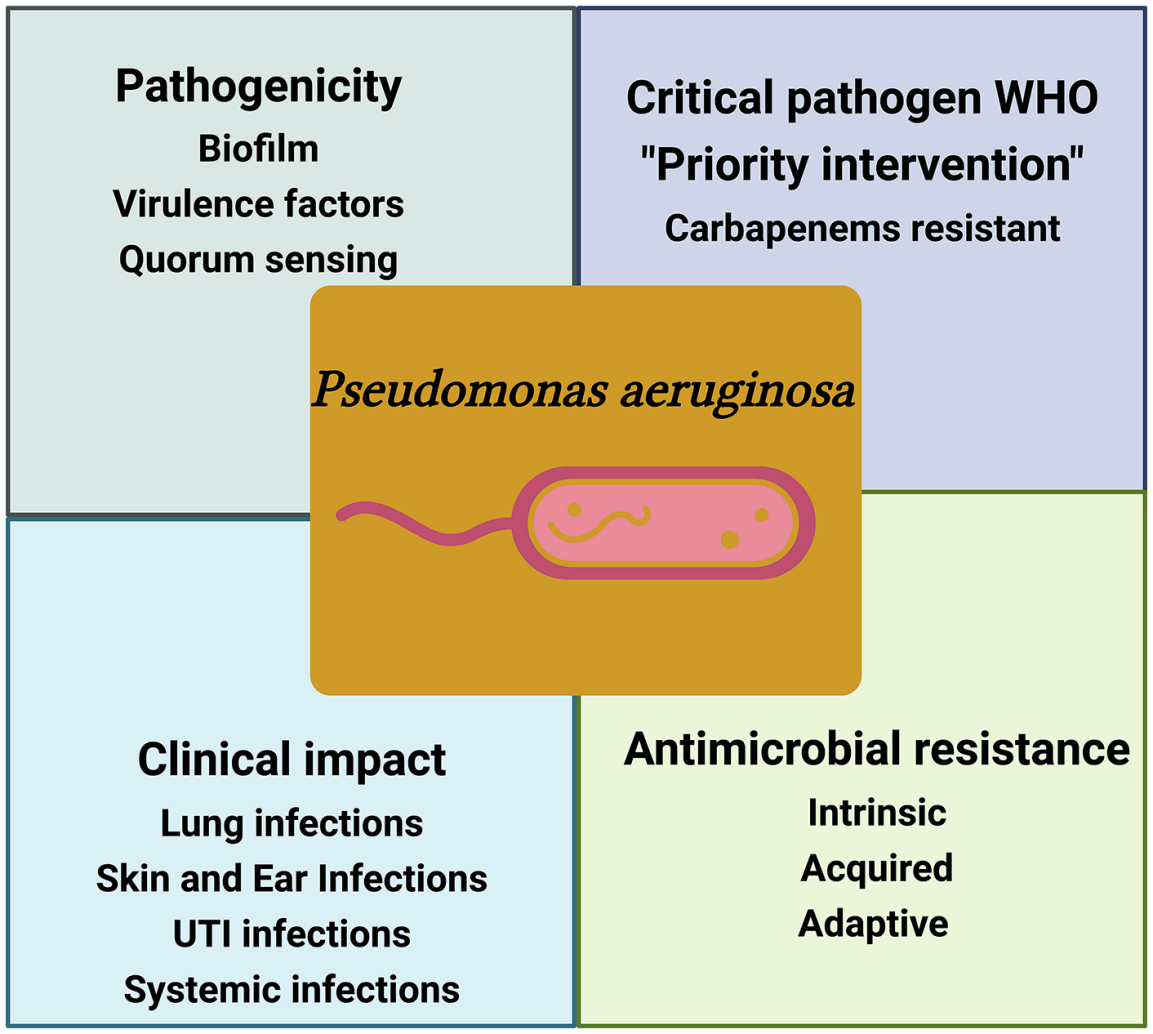
Schematic illustration of the key factors of *P. aeruginosa* pathogencity. WHO-designated critical pathogen, *P. aeruginosa*, exhibits intricate antimicrobial resistance, posing a global health threat and causing diverse clinical infections, the figure was created with biorender.

*Pseudomonas aeruginosa* stands at the forefront of antibiotic resistance, notably earning a place on the WHO list of “priority pathogens” (Balakrishnan, 2022). These pathogens have posed a critical therapeutic challenge by demonstrating resistance to multiple antimicrobials, including imipenem, meropenem, and 3rd generation cephalosporins, which are currently the most effective drugs for combatting MDR bacteria. *P. aeruginosa* is part of the notorious group of “ESKAPE” pathogens, named for *Enterococcus faecium, Staphylococcus aureus, Klebsiella pneumoniae, Acinetobacter baumannii, Pseudomonas aeruginosa*, and *Enterobacter* spp., which are deemed of the highest concern due to their urgent need for new antibiotics (Mulani et al., 2019) (Figure 2).

The clinical impact of *P. aeruginosa* infections is profound, particularly among vulnerable populations such as critically ill patients, immunocompromised individuals, and the very young and elderly. It is considered the 4th most frequently isolated nosocomial pathogen and one of the life-threatening bacteria showing a high mortality rate in immunocompromised patients (Hafiz et al., 2023). The impact of *P. aeruginosa* and its significance has been recognized early. Data from the National Nosocomial Infections Surveillance System (NNISS) between 1986 and 2003 revealed alarming statistics. *P. aeruginosa* ranked as the second most common cause of pneumonia (18.1%), the third most common cause of UTIs (16.3%), and the 8th most frequently isolated pathogen in BSIs (3.4%) (Gaynes and Edwards, 2005). However, what adds to the gravity of the situation is the disturbing surge in the proportion of resistant *P. aeruginosa* isolates over the subsequent years. Studies from China have reported a 28.7% prevalence of *P. aeruginosa-related* infections in tertiary care settings ranking second to *Acinetobacter baumannii* in its frequency of isolation (Oliveira et al., 2015). Other observational point-prevalence found that *P. aeruginosa* represented 16.2% of infections in ICU patients and was the cause of 23% of all ICU-acquired infections, with a respiratory source being the most common site of *P. aeruginosa* infection (Vincent et al., 2020). Resistance to key antibiotics like carbapenems, quinolones, and 3rd generation cephalosporins surged by significant margins. A particular strain known as the “pan drug-resistant” (PDR) *P. aeruginosa* was isolated in 2006, rendering it resistant to virtually all available antimicrobial agents, including cefepime, ceftazidime, imipenem, meropenem, piperacillin-tazobactam, ciprofloxacin, and levofloxacin (Wang et al., 2006). This emergence of “intractable” infectious agents heralds the drastic consequences of the antibiotic resistance era, as emphasized by numerous public health organizations.

Multiple studies conducted in subsequent years continued to demonstrate *P. aeruginosa* resistance profile. Resistance rates to various antimicrobial agents, including cefepime, piperacillin, ciprofloxacin, levofloxacin, gentamicin, and amikacin, displayed worrying trends. Eastern and southern parts of Europe, particularly Germany, Hungary, and Slovakia, reported high resistance percentages in *P. aeruginosa* (European Centre for Disease Control and Prevention, 2015). By 2015, resistance to third and 4th generation cephalosporins like ceftazidime and cefepime had also escalated. A study in 2016 noted *P. aeruginosa* involvement in nosocomial infections, with UTIs being predominant among patients. The global scope of the *P. aeruginosa* resistance problem is evident from studies conducted in India, where drug resistance rates to various antibiotics were reported, reflecting an alarming upward trend (Choudhuri et al., 2017). The sensitivity of *P. aeruginosa* strains to colistin, one of the last-resort antibiotics, remained relatively high, while susceptibility to other commonly used anti-pseudomonal drugs diminished. The prevalence of MDR strains further complicated treatment options, and high resistance levels against antibiotics like quinolones and aminoglycosides were noted (Horcajada et al., 2019). According to the European Centre for Disease Prevention and Control’s annual report, 18.7% of all *P. aeruginosa* isolates were carbapenem-resistant and 13.4% were resistant to three or more antimicrobial classes (ECDC, 2021). Additionally, reports from veterinary medicine and agriculture pointed to a challenging scenario, with resistance observed even in carbapenems, considered among the most potent antibiotics (Elfadadny et al., 2023b). Given the continuing advance of antimicrobial resistance within *P. aeruginosa* strains, there is an escalating urgency for the development of innovative therapeutic options. The appearance and widespread dissemination of PDR strains emphasize the severity of this issue. The collaborative endeavors of prominent global health organizations, including the Centers for Disease Control and Prevention (CDC), Infectious Disease Society of America (IDSA), World Economic Forum, and the WHO, emphasize the profound magnitude of the antimicrobial resistance challenge and the imperative for a worldwide response.

*Pseudomonas aeruginosa* is indebted its remarkable virulence to a formidable array of factors that enable it to initiate and sustain infections, particularly in immunocompromised individuals (Qin et al., 2022). These virulence factors organize a coherence of pathogenicity, enhancing the bacterium’s capacity to colonize, invade, and persist within host tissues. A closer examination of these factors reveals the intricate mechanisms by which *P. aeruginosa* inflicts damage. **Alginate and biofilm formation**: in patients with chronic respiratory infections, *P. aeruginosa* produces a remarkable exopolysaccharide known as alginate. This polysaccharide serves as an adhesive, allowing the bacterium to adhere to surfaces, and provides a protective shield against inhospitable environmental conditions. *P. aeruginosa* also secretes alginate lyase, an enzyme capable of cleaving alginate into shorter oligosaccharide units (Boyd and Chakrabarty, 1995). This dynamic interplay between alginate production and degradation plays a pivotal role in the pathogenicity of *P. aeruginosa*. **Adhesion and twitching motility**: *P. aeruginosa* initiates infection by binding to gangliosides on host epithelial surfaces. This interaction is facilitated by LPS and bacterial adhesins, including type-IV pili and flagella. Type-IV pili serve to enable adhesion and facilitate “twitching motility.” This unique form of movement allows the bacterium to traverse host cell surfaces, significantly enhancing biofilm development—a critical aspect of *P. aeruginosa*’s pathogenicity (Kühn et al., 2021). **Type III secretion system (T3SS)**: upon attachment to host cells, *P. aeruginosa* activates its Type III secretion system creating a channel through which cytotoxic effector proteins are injected into the host cell’s cytosol. Four types of toxins are produced, including ExoS, ExoT, ExoU, and ExoY (Hardy et al., 2021). These toxins wield diverse capabilities, such as GTPase-activating proteinase (GAP) activity, ADP-ribosyl transferase activity (ADPRT), adenylate cyclase activity, and even membrane phospholipid cleavage, an effect seen in ExoU potent phospholipase A2 (PLA2) activity. These toxins collectively initiate inflammation and subvert host cell functions. **Exotoxin A**: *P. aeruginosa* secretes Exotoxin A, an ADPRT that disrupts protein synthesis by inhibiting the host elongation factor 2 (EF2). This disruption results in cell death, further exacerbating pathogenic effects (Michalska and Wolf, 2015). **Lipases and phospholipases**: lipases and phospholipases produced by *P. aeruginosa* contribute to pathogenicity by dissolving surfactant lipids and host cell membrane phospholipids. This destructive action compromises host cell integrity, aiding bacterial invasion (Rosenau et al., 2010). **Pyocyanin and pyoverdine**: the blue-green pigment pyocyanin plays a pivotal role in pathogenicity by inducing oxidative stress within host cells. It disrupts host catalase and the electron transport system (ETS), suppressing phagocytosis (Hall et al., 2016). Pyoverdine also interferes with host electron transport pathways and the redox cycling system, impairing immune system function (Durán et al., 2022). **Type VI secretion system (T6SS)**: comprising H1, H2, and H3 variants, the T6SS enhances *P. aeruginosa*’s survival and virulence. H1-T6SS plays a role in antimicrobial activity, while H2-and H3-T6SS enable interactions with both prokaryotic and eukaryotic cells (Lesic et al., 2009). **Elastases**: two types of elastases, LasA and LasB, contribute to the pathogenesis of *P. aeruginosa*. LasA hydrolyzes the penta-glycine bridge crucial for peptidoglycan stabilization in the cell wall. In contrast, LasB is responsible for the opsonization of lung surfactant proteins A and D (Cigana et al., 2021).

Understanding the genetic similarity and dissemination of *P. aeruginosa* clones is paramount for both localized infection control and global public health surveillance (Dos Santos et al., 2023). Various methods with varying costs, labor intensity, and discriminatory power have been employed to assess genetic relatedness among *P. aeruginosa* strains. These methods serve as essential tools for not only managing local outbreaks but also identifying successful international clones. Traditional methods such as serotyping and phage typing rely on the differential sensitivity of isolates to standardized bacteriophages (Waters et al., 2012). These techniques, while less discriminatory, are valuable for managing local outbreaks. Pyocin typing involves the characterization of strains based on their susceptibility to pyocins, which are antibacterial proteins produced by *P. aeruginosa* (Michel-Briand and Baysse, 2002). This method offers moderate discriminatory power. Molecular techniques like Pulse-Field Gel Electrophoresis (PFGE), Field-Inversion Gel Electrophoresis (FIGE), and Random Amplified Polymorphic DNA Polymerase Chain Reaction (RAPD-PCR) (Hematzadeh and Haghkhah, 2021), provide higher discriminatory power and are often used in local outbreak investigations. Oligonucleotide microarrays allow for simultaneous analysis of multiple genes, offering more comprehensive strain characterization (Wagner et al., 2003). Additionally, Multi-Locus Sequence Typing (MLST) involves sequencing specific housekeeping genes to identify genetic variation among isolates (Curran et al., 2004). It provides a standardized nomenclature for strain comparison. Whole-genome sequencing (WGS) offers the highest discriminatory power and has become a gold standard in identifying international clones. It enables the comprehensive analysis of an organism’s entire genetic makeup (Madaha et al., 2020).

Currently, three major international MDR clones have emerged as global threats: ST111, ST175, and ST235 (del Barrio-Tofiño et al., 2020). ST111 (serotype O12) and ST235 (serotype O11) have demonstrated widespread distribution across continents, while ST175 (serotype O4) has primarily been confined to European countries, especially in Spain and France (Pea et al., 2015; del Barrio-Tofiño et al., 2020). ST235 clones are particularly noteworthy due to their high virulence, driven by the presence of ExoU, a potent cytotoxin. These isolates pose significant therapeutic challenges as they exhibit multidrug resistance. Interestingly, the fitness burden associated with maintaining MDR in ST235 clones appears to be comparatively lower, possibly contributing to their successful dissemination. WGS has further allowed for the categorization of *P. aeruginosa* isolates into three distinct resistotypes: PAO1, PA14, and PA7 (Reboud et al., 2017). PAO1 and PA14 are characterized by possessing the T3SS and the corresponding effector toxins, ExoS (PAO1) and ExoU (PA14) (Reboud et al., 2017). In contrast, PA7 lacks T3SS and employs the Type VI secretion system to secrete ExlA exolysin, damaging surrounding tissue cells. Interestingly, the carriage of the *exoU* gene in *P. aeruginosa* isolates has been associated with resistance to aminoglycosides and fluoroquinolones, suggesting a possible link between virulence and antibiotic resistance.

## 4 Types of resistance in *Pseudomonas aeruginosa*

**Figure 3:**
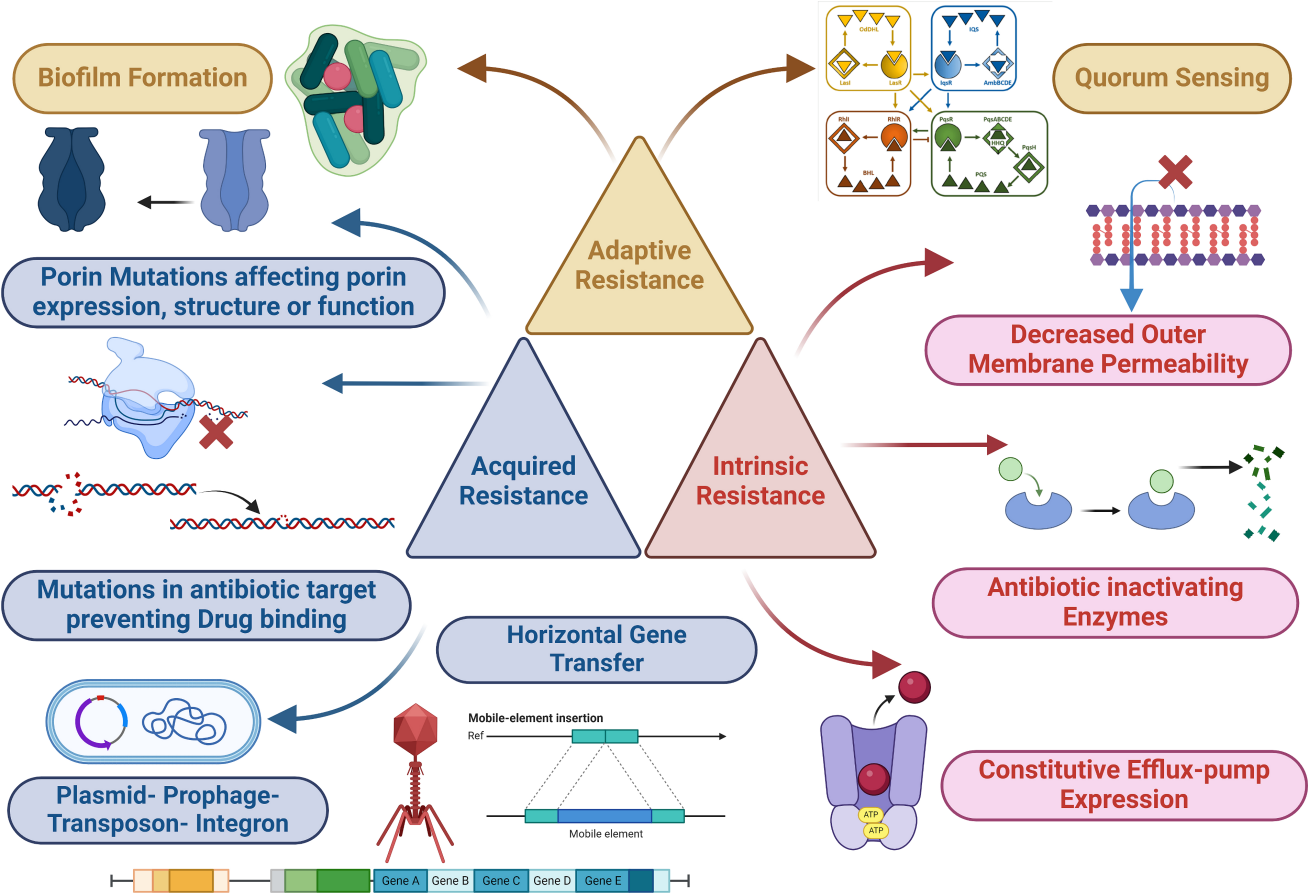
Mechanism of antimicrobial resistance (acquired, intrinsic, and adaptive) of *P. aeruginosa*. Parts of the figure was edited from (Schütz and Empting, 2018), the figure was created with biorender.

*Pseudomonas aeruginosa* deploys a spectrum of resistance mechanisms challenging traditional antibiotic therapies. Intrinsic resistance, a foundational trait encoded in its genome, involves factors like low outer membrane permeability, Mex-type efflux pumps, and AmpC β-lactamase. These elements collectively establish a basal resistance level present across all strains. Acquired resistance includes mutations influencing antibiotic targets and the acquisition of modifying enzymes or resistance plasmids. This adaptability enables the bacterium to evolve swiftly, undermining the efficacy of conventional antibiotics. Intriguingly, *P. aeruginosa* exhibits adaptive resistance mechanisms, responding dynamically to environmental stimuli. This feature allows the bacterium to adjust its resistance profile based on host factors and signal molecules, further complicating treatment strategies. The interplay of these resistance mechanisms contributes to the development of MDR strains, posing a serious threat to public health (Figure 3). Clarification of the complexities of these mechanisms is imperative for the development of innovative therapies that can effectively combat the rising challenges posed by *P. aeruginosa* infections.

### 4.1 Acquired resistance

#### 4.1.1 Mutation

Mutation-driven resistance in *P. aeruginosa* is a complex phenomenon involving various mechanisms that enable the bacterium to withstand the effects of antimicrobial drugs. These mutations affect critical aspects of bacterial biology, resulting in several resistance mechanisms (Serwecińska, 2020). **Enhanced β-lactamase production**: mutation-induced disruptions in the DNA oxidative repair system can increase mutation rates in *P. aeruginosa*. This, in turn, leads to enhanced production of β-lactamase enzymes, which can break down β-lactam antibiotics. Additionally, mutations can cause the overexpression of efflux pumps, such as MexCD-OprJ, which actively remove antibiotics from bacterial cells, reducing the drugs’ effectiveness. The primary mechanism driving β-lactam resistance, often caused by mutations, frequently involves the overproduction of the chromosomal cephalosporinase known as *ampC* (Lorusso et al., 2022). This process engages a complex network of genes associated with intricate regulatory pathways responsible for recycling cell wall components. Among these genes, mutational inactivation of *dac*B, which encodes the non-essential penicillin-binding protein (PBP) PBP4, and *amp*D, responsible for encoding N-acetyl-muramyl-l-alanine amidase, are the most common causes leading to *ampC* activation and consequent β-lactam resistance (Zamorano et al., 2010). Interestingly, the inactivation of PBP4 has been shown to activate the BlrAB/CreBC regulatory system, further intensifying the levels of resistance (Moya et al., 2009). Moreover, specific point mutations that induce a conformational change in the transcriptional regulator AmpR, thereby upregulating *ampC* and promoting β-lactam resistance, have been observed in clinical strains. Examples of such mutations include D135N and the R154H mutation, which is associated with the widespread MDR/XDR ST175 high-risk clone (Juan et al., 2017). Furthermore, numerous other genes, including those coding for various amidases (AmpDh2 and AmpDh3), different PBPs (such as PBP5 and PBP7), lytic transglycosylases (such as SltB1 and mltB), MPL (UDP-N-acetylmuramate: L-alanyl-γ-D-glutamyl-meso-diaminopimelate ligase), and NuoN (NADH dehydrogenase I chain N), have been shown to enhance *ampC* expression (Ropy et al., 2015). In addition to *ampC* overexpression, recent studies have unveiled alternative pathways to β-lactam resistance development. This includes the emergence of novel combinations of β-lactam-β-lactamase inhibitors such as ceftolozane/tazobactam and ceftazidime/avibactam due to mutations that induce structural modifications in AmpC (Haidar et al., 2017). To date, more than 500 Pseudomonas Derived Cephalosporinase (PDC) variants, https://arpbigidisba.com/pseudomonas-aeruginosa-derived-cephalosporinase-pdc-database/, have been characterized, including those linked to enhanced resistance against ceftolozane/tazobactam and ceftazidime/avibactam. **Porin mutations and reduced membrane permeability**: *P. aeruginosa* relies on porin proteins, like OprD the second major porin protein and one of the most well-characterized in *P. aeruginosa*, to create channels within its membrane for the entry of hydrophilic antibiotics (Welte et al., 1995). Mutations affecting porin expression or function can reduce bacterial membrane permeability, resulting in increased antibiotic resistance. OprD deficiency, for instance, confers high-level resistance to carbapenem antibiotics, particularly imipenem (Fang et al., 2014). A study conducted in southern China investigated 61 clinical isolates of *P. aeruginosa* that exhibited resistance to imipenem. Their findings indicated that among these isolates, 50 had mutations leading to the disruption of OprD (Fang et al., 2014). These mutations included frameshift mutations or the presence of premature stop codons. Additionally, 5 isolates showed reduced *Opr*D expression, while in 6 isolates, *Opr*D was undetectable through PCR analysis. Furthermore, the study conducted a functional analysis, revealing that loops 2 and 3 within the OprD protein served as entrance and binding sites for imipenem. **Efflux pump overexpression and regulatory mutations**: efflux pumps play a crucial role in antibiotic resistance by pumping antibiotics out of bacterial cells. Mutations in regulatory genes like *mex*R, *nal*B, *nal*C, or *nal*D can disrupt the normal control of efflux pump expression. Consequently, this leads to the overexpression of efflux pumps such as MexAB-OprM, reducing susceptibility to various antibiotics, including β-lactams and fluoroquinolones (Pang et al., 2019). Mutations in the *mex*Z gene can also induce overexpression of MexXY-OprM, increasing resistance to aminoglycosides, β-lactams, and fluoroquinolones. **Modifications in antibiotic targets**: *P. aeruginosa* can modify antibiotic targets through mutations. For example, mutations in genes encoding DNA gyrase (*gyrA* and *gyrB*) and topoisomerase IV (*parC* and *parE*) can alter the binding affinity of these proteins to quinolone antibiotics, reducing susceptibility to quinolones (Hooper and Jacoby, 2016). Additionally, mutations can affect ribosomal proteins (30S ribosomal subunit), leading to high resistance to aminoglycosides that target protein translation. Modifications in PBPs can increase resistance to β-lactam antibiotics. Resistance to polymyxin in *P. aeruginosa* has been demonstrated to correlate with alterations in the partner molecule for polymyxin binding-LPS (Gutu et al., 2013). This involves the addition of 4-amino-L-arabinose (L-Ara4N) to the phosphate groups located within the lipid A portion of LPS (Pang et al., 2019). **Structural modifications of antibiotic targets**: resistance to novel combinations of β-lactam-β-lactamase inhibitors can result from mutations leading to structural modifications of AmpC. Moreover, diverse AmpC variants have been associated with high-level cephalosporin resistance. Additionally, mutations in genes encoding proteins like PBP3 (encoded by *fts*I) contribute to β-lactam resistance. Specific mutations in PBP3 have been associated with β-lactam resistance in clinical strains (Sethuvel et al., 2023).

#### 4.1.2 Horizontal gene transfer

Horizontal gene transfer plays a pivotal role in the acquisition of antibiotic resistance genes by *P. aeruginosa*, enabling it to adapt to various environmental challenges. Antibiotic resistance genes can be harbored on diverse genetic elements such as plasmids, transposons, integrons, and prophages (Michaelis and Grohmann, 2023). These genes can be acquired by *P. aeruginosa* through horizontal gene transfer, either from closely related bacterial species or distant ones. Integrons, which are genetic elements specialized in inserting mobile gene cassettes through site-specific recombination, have emerged as critical players in the dissemination of antibiotic resistance within *P. aeruginosa* populations. These integrons serve as repositories for a wide array of antibiotic resistance determinants (Bhat et al., 2023). Horizontal gene transfer mechanisms include transformation, transduction, and conjugation. *P. aeruginosa* has demonstrated the ability to acquire genes conferring resistance to aminoglycosides and β-lactam antibiotics. Notably, various metallo-beta-lactamase (MBL) genes, such as IMP, VIM, SPM, GIM, NDM, and FIM, which encode enzymes capable of hydrolyzing a broad spectrum of β-lactam antibiotics, have been identified in *P. aeruginosa* (Potron et al., 2015). These MBL genes are often associated with genetic elements like integrons and plasmids. Furthermore, integrons have been found to harbor multiple antibiotic-resistance genes simultaneously. For example, aminoglycoside resistance genes *aacA29a* and *aacA29b* have been identified within class I integrons in clinical *P. aeruginosa* isolates, situated at the 5′ and 3′ ends of the carbapenem-hydrolyzing β-lactamase VIM-2 gene cassette, respectively. in addition, it is important to note that many resistance genes in *P. aeruginosa* are located within resistance islands rather than plasmids (Magnet et al., 2003). Unlike some plasmids that are readily transferable to *E. coli*, many Pseudomonas spp. plasmids have a narrow host range. These plasmids have been classified using a separate incompatibility typing system known as IncP-1 to IncP-13 (Xiong et al., 2013). IncP-2 plasmids, historically common in *P. aeruginosa*, were typically large and carried tellurite resistance in addition to antibiotic resistance. More recently, various plasmids from *P. aeruginosa*, including those from carbapenem-resistant strains, have been sequenced, revealing their role in the dissemination of resistance genes, particularly carbapenemase genes including *bla*IMP-9, its variant *bla*IMP-45, or *bla*VIM-2. These plasmids often carry class 1 integrons within Tn21 subfamily transposons (Xiong et al., 2013).

### 4.2 Intrinsic resistance

#### 4.2.1 Efflux system resistance

Bacterial efflux systems are categorized into five major families: Resistance Nodulation Division (RND), ATP Binding Cassette (ABC), Major Facilitator Superfamily (MFS), Small Multidrug Resistance (SMR), and Multidrug & Toxic Compound Extrusion (MATE) (Huang et al., 2022). Among these, the RND family, characterized by its cytoplasmic membrane transporters, periplasmic linker proteins, and outer membrane porin channel proteins, holds a pivotal role in antibiotic resistance, particularly in *P. aeruginosa*. These efflux systems function as a defense mechanism by expelling harmful substances from bacterial cells (Poole, 2011). The RND family of efflux systems consists of two essential components: multidrug efflux (Mex) and outer membrane porin (Opr) (Qin et al., 2022). These components work in synergy to facilitate the efflux of various toxic compounds, including antibiotics. Within the RND family, the MexXY efflux system is a key player. It encodes two crucial proteins, MexY and MexX, which work together to expel toxic substances from the bacterial cell (Dreier and Ruggerone, 2015). This efflux system has a broad substrate range, allowing it to efficiently pump out a variety of antibiotics and other harmful compounds. MexAB-OprM is renowned for its resistance against multiple antibiotics, including ticarcillin, broad-spectrum cephalosporins, and β-lactams. In clinical isolates, it’s frequently associated with β-lactam resistance. A combination of efflux systems, including MexAB-OprM, MexCD-OprJ, and MexXY-OprM, contributes to carbapenem resistance (Riera et al., 2011). Although efflux plays a role, it often acts alongside other resistance mechanisms. Besides *P. aeruginosa*, other bacteria like *Burkholderia pseudomallei* and *E. coli* employ three-component RND-type aminoglycoside efflux systems in their resistance strategies. Notably, the expression of MexXY can be upregulated in mutants when exposed to antibiotics, enhancing their resistance compared to wild-type strains. Wild-type strains of *P. aeruginosa* inherently possess resistance to specific antibiotic classes, such as tetracyclines, aminoglycosides, glycylcyclines, and erythromycin. However, the MexXY efflux system extends this resistance to a wide array of antibiotics, including lincomycin, macrolides, fluoroquinolones, chloramphenicol, β-lactams, and novobiocin. This broad substrate range contributes significantly to the remarkable antibiotic resistance observed in *Pseudomonas*. The expression of the MexXY gene within the RND family is tightly regulated by the mexZ repressor, which belongs to the tetracycline repressor protein (TetR) and AcrR repressor protein family (Fujiwara et al., 2022). This ensures that the efflux system responds appropriately to the presence of antibiotics. Overexpression of these pumps has been documented in various clinical strains of *P. aeruginosa*, substantially amplifying bacterial antibiotic resistance.

#### 4.2.2 Outer membrane impermeability resistance

In the context of antibiotic resistance, overcoming the protective fortress of the bacterial cell membrane is a challenge faced by antibiotics. These resilient pathogens, like *P. aeruginosa*, deploy an array of defense mechanisms that antibiotics must navigate. Distinct antibiotic classes employ specific strategies to combat *P. aeruginosa*. Aminoglycoside antibiotics, such as tobramycin, gentamicin, and amikacin, disrupt bacterial protein synthesis by binding to ribosomal 30S subunits (Krause et al., 2016). Meanwhile, quinolone antibiotics, including ciprofloxacin and levofloxacin, obstruct DNA replication through inhibition of DNA gyrase and topoisomerase IV (Hooper and Jacoby, 2016). The β-lactam antibiotics, encompassing penicillin, cephalosporin, carbapenem, and monobactam, hinder bacterial cell wall biosynthesis by targeting PBPs, pivotal enzymes in peptidoglycan synthesis (Cho et al., 2014). Polymyxins, exemplified by polymyxin B and polymyxin E (colistin), are polypeptide antibiotics that bind to the LPS on the outer membrane of Gram-negative bacteria (Slingerland et al., 2022). This binding enhances cell membrane permeability, facilitating greater antibiotic uptake. Remarkably, polymyxins execute their bactericidal effects by inducing a hydroxyl radical-mediated cell death pathway. For β-lactams and quinolones, penetration through porin channels within the bacterial cell membrane is the pathway of choice. In contrast, aminoglycosides and polymyxins employ a different strategy, interacting with bacterial LPS on the outer membrane of Gram-negative bacteria to enhance their uptake (Prajapati et al., 2021). The outer membrane of Gram-negative bacteria, including *P. aeruginosa*, acts as a selective barrier, complicating antibiotic penetration. This outer membrane is an asymmetric bilayer containing phospholipids and LPS. Porins, forming β-barrel protein channels, are scattered within this structure. LPS itself comprises lipid A, an oligosaccharide core, and O antigen. Notably, organic compounds like citric and lactic acid act as chelating agents, disrupting the oligosaccharide core by binding to Mg2+ cations in the LPS molecule. This interaction alters the bacterial cell wall’s permeability, adding an extra layer of resistance. Within *P. aeruginosa*, a diverse family of porins contributes to regulating permeability. OprF serves as the major non-specific porin, while OprB, OprD, OprE, OprO, and OprP are specific porins. OprC and OprH are categorized as gated porins, and the efflux porins include OprM, OprN, and OprJ (Qin et al., 2022). Understanding these intricate mechanisms offers insights into the multifaceted strategies employed by *P. aeruginosa* to resist antibiotic intrusion.

#### 4.2.3 Enzyme modification

*P. aeruginosa* wields a formidable weapon, antibiotic-inactivating enzymes. These enzymes possess the ability to dismantle or modify antibiotics, rendering them powerless. This intrinsic defense mechanism presents a significant hurdle for clinicians combating *P. aeruginosa* infections. One of the most critical classes of antibiotic-inactivating enzymes in *P. aeruginosa* is β-lactamases. β-lactam antibiotics, including penicillin and cephalosporins, target bacterial cell walls by inhibiting PBP. *P. aeruginosa* has honed its β-lactamase production to counteract these antibiotics by hydrolyzing the b-lactam ring that is present in b-lactam antibiotics. β-lactamases are categorized into four classes: A, B, C, and D, depending on their amino acid sequences (Bush and Bradford, 2016). Classes A, C, and D utilize an active site serine to hydrolyze β-lactams, whereas class B β-lactamases are metalloenzymes requiring zinc ions for this process. *P. aeruginosa* produces class C β-lactamases, which have been demonstrated to hinder antipseudomonal cephalosporins, a subgroup of β-lactam antibiotics. Some *P. aeruginosa* strains go a step further by generating extended-spectrum-β-lactamases (ESBLs), primarily falling into class A. Interestingly, OXA-type ESBLs, recognized for their ability to hydrolyze oxacillin, are classified under enzyme class D and were initially identified in *P. aeruginosa* isolates (Castanheira et al., 2021). These ESBLs confer a high level of resistance to a wide array of β-lactam antibiotics, encompassing penicillins, cephalosporins, and aztreonam. Additionally, AmpC β-lactamase, an Ambler class C enzyme, encoded by the chromosomal *bla*Amp*C* gene, is a central player in *P. aeruginosa’s* resistance strategy. AmpC is constitutively produced and can be induced further by aminopenicillins and cephalosporins. It hydrolyzes the β-lactam ring, effectively inactivating β-lactam antibiotics. Metallo β-lactamases (MBLs) are categorized as Ambler class B enzymes, distinct from the serine-based hydrolytic system. Notably, MBLs feature zinc in their active sites. In *P. aeruginosa*, the most clinically significant MBLs are the Verona integron-encoded MBLs (VIM) and the active-on-imipenem type MBLs (IMP) (Tooke et al., 2019). These enzymes are frequently situated on integrons, often alongside other resistance genes, and confer high-level resistance to carbapenem antibiotics.

Aminoglycoside antibiotics, frequently employed in the treatment of *P. aeruginosa* infections, face a formidable challenge in the form of enzymatic modification-based resistance mechanisms employed by this bacterium. *P. aeruginosa* employs an array of enzymes with specific functions in this process. Aminoglycoside Phosphotransferases (APHs) play a crucial role by modifying aminoglycosides like kanamycin, neomycin, and streptomycin. They achieve this by transferring a phosphoryl group, effectively rendering these antibiotics inactive. In parallel, Aminoglycoside Acetyltransferases (AACs) enter the scene, transferring an acetyl group to amino groups at positions 3′ and 6′ of aminoglycosides. This acetylation process leads to the inactivation of a range of aminoglycosides, including gentamicin, tobramycin, netilmicin, kanamycin, and amikacin (Ramirez and Tolmasky, 2010). Furthermore, Aminoglycoside Nucleotidyltransferases (ANTs) add another layer of defense by transferring an adenylyl group to either the amino or hydroxyl group of aminoglycosides, effectively conferring resistance against antibiotics such as gentamicin, amikacin, and tobramycin (Ramirez and Tolmasky, 2010).

### 4.3 Adaptive resistance

#### 4.3.1 Biofilm formation and quorum sensing mediated resistance

Biofilm formation is a remarkable survival strategy employed by *P. aeruginosa*. In this complex process, *P. aeruginosa* communities adhere to surfaces and are encapsulated within a matrix of extracellular polymeric substances (EPSs), which includes exopolysaccharides, proteins, metabolites, and extracellular DNA (eDNA). *P. aeruginosa* biofilms are characterized by a sophisticated matrix composed of various EPS components, including complex polysaccharides, proteins, lipids, and eDNA (Thi et al., 2020). This matrix provides structural integrity to the biofilm and offers several critical advantages to the bacteria within it. *Immune System Evasion*: Biofilms provide physical protection from the host immune system. The matrix acts as a barrier, preventing immune cells from reaching and eliminating the bacteria (Vestby et al., 2020). *Antimicrobial Resistance*: Bacteria within biofilms exhibit a significantly higher resistance to antimicrobial agents, including antibiotics. This resistance arises from reduced antibiotic penetration and decreased metabolic activity within biofilm-associated cells (Singh et al., 2017). *Water Retention and Desiccation Tolerance*: Biofilms retain water, which helps the bacteria survive in arid or hostile environments. This property contributes to the resilience of biofilm communities (Secor et al., 2015). *Nutrient Storage and High Enzymatic Activity*: Biofilms can efficiently sorb and store nutrients, supporting bacterial growth. They also exhibit heightened extracellular enzymatic activity, aiding in resource utilization. *Adhesion and Virulence*: The biofilm matrix facilitates adhesion to infection sites, promoting persistent colonization (Limoli et al., 2015).

Biofilm-associated infections pose significant clinical challenges. *P. aeruginosa’s* biofilm formation results in a profound increase in antibiotic tolerance, making treatment difficult. Furthermore, the diffusion barrier created by the biofilm matrix limits antibiotic exposure to only the superficial layers of the biofilm, rendering deep-seated infections highly resistant to treatment. *P. aeruginosa* is renowned for its biofilm-forming capabilities. These biofilms can develop in various environments, including the anaerobic conditions found in the lungs of cystic fibrosis (*CF*) patients (Valentin et al., 2022). Biofilm formation by *P. aeruginosa* is a finely tuned process regulated by various factors: *Quorum Sensing Systems*: *P. aeruginosa* employs multiple quorum sensing systems, including LasI-LasR, RhlI-RhlR, and PQS-MvfR, which contribute to the formation of mature and differentiated biofilms (Ambreetha and Singh, 2023). *Two-Component Regulatory Systems*: The GacS/GacA and RetS/LadS two-component systems play crucial roles in regulating biofilm formation. GacA promotes biofilm formation, while RetS inhibits it (Mikkelsen et al., 2011). *Exopolysaccharides*: Alginate, Pel, and Psl are exopolysaccharides produced by *P. aeruginosa*, stabilizing the biofilm structure (Colvin et al., 2012). *eDNA*: eDNA serves as a vital component of the biofilm matrix. It contributes to initial cell–cell adhesion, protects the biofilm from detergents, and influences aminoglycoside resistance (Wei and Ma, 2013). *Cyclic di-GMP (c-di-GMP)*: Intracellular levels of c-di-GMP play a crucial role in biofilm formation. High c-di-GMP levels are associated with biofilm formation, whereas low levels correspond to planktonic growth (Park and Sauer, 2022).

During biofilm formation, *P. aeruginosa* undergoes various physiological changes. In *CF* chronic infections, *P. aeruginosa* strains switch to a mucoid phenotype, characterized by increased alginate production. Additionally*, P. aeruginosa* initially relies on its flagellum for motility but downregulates flagellum expression after surface attachment to evade immune detection. Recent investigations have unveiled critical insights into the formidable antibiotic resistance mechanisms at play within *P. aeruginosa* biofilms. In a study by Sadovskaya and colleagues in 2010 (Sadovskaya et al., 2010), a family of cyclic glycerophosphorylated β-(1, 3)-glucans secreted by *P. aeruginosa* was identified within the extracellular matrix of biofilms. These glucans were found to interact with and sequester kanamycin, a frequently used antibiotic, rendering it less effective. Additionally, research conducted shed light on novel genes present in the *P. aeruginosa* clinical isolate PA14. While these genes did not directly influence biofilm formation, they played a crucial role in enhancing biofilm-specific antibiotic resistance. The *ndvB* gene, for instance, encoded a vital glucosyltransferase responsible for synthesizing periplasmic cyclic-β-(1, 3)-glucans, which physically captured tobramycin, preventing it from reaching its site of action. Another gene cluster participated in the creation of a novel efflux pump within *P. aeruginosa*. Its deletion resulted in reduced resistance of the bacterium to antibiotics like gentamicin and ciprofloxacin within the biofilm. Lastly, the *tssC1* gene, associated with the type VI secretion system in *P. aeruginosa*, exhibited heightened expression in biofilms. When *tssC1* was deleted, it led to diminished resistance to antibiotics commonly employed in the treatment of *P. aeruginosa* infections, including tobramycin, gentamicin, and ciprofloxacin (Zhang et al., 2011).

Quorum sensing (QS) is an intriguing communication system used by *P. aeruginosa* to coordinate its behavior. In this system, *P. aeruginosa* relies on the release of specific signaling molecules known as autoinducers to communicate with other bacteria. Notably, *P. aeruginosa* employs four distinct QS pathways to achieve this communication. Two of these pathways involve LasR and LasI, as well as RhlR and RhlI. LasI, part of the LasR/I system, produces a molecule called 3O-C12-HSL. This molecule plays a vital role in the QS process (Kanak et al., 2023). It binds to LasR, which triggers the expression of LasI and leads to the production of virulence factors. Additionally, it contributes to the formation of biofilms. On the other hand, the Rhl system involves RhlI, which synthesizes another signaling molecule called C4-HSL. When C4-HSL binds to RhlR, it activates RhlI, initiating the expression of genes responsible for virulence factors and biofilm components. These two systems, LasR/I and RhlR/I, are interconnected, and their activities are influenced by a molecule called the Pseudomonas quinolone signaling system (PQS), which acts as a bridge between them. Las and Rhl systems regulate the synthesis of PQS, and in turn, regulate the activity of RhlR and RhlI (Venturi, 2006). Understanding this intricate web of quorum sensing in *P. aeruginosa* provides valuable insights into how this bacterium causes disease and becomes resistant to antibiotics. Researchers are actively exploring innovative approaches to combat *P. aeruginosa* infections, focusing on anti-virulence strategies that target QS and the inhibition of biofilm formation.

## 5 Innovative therapies for resistance in *Pseudomonas aeruginosa*

**Figure 4:**
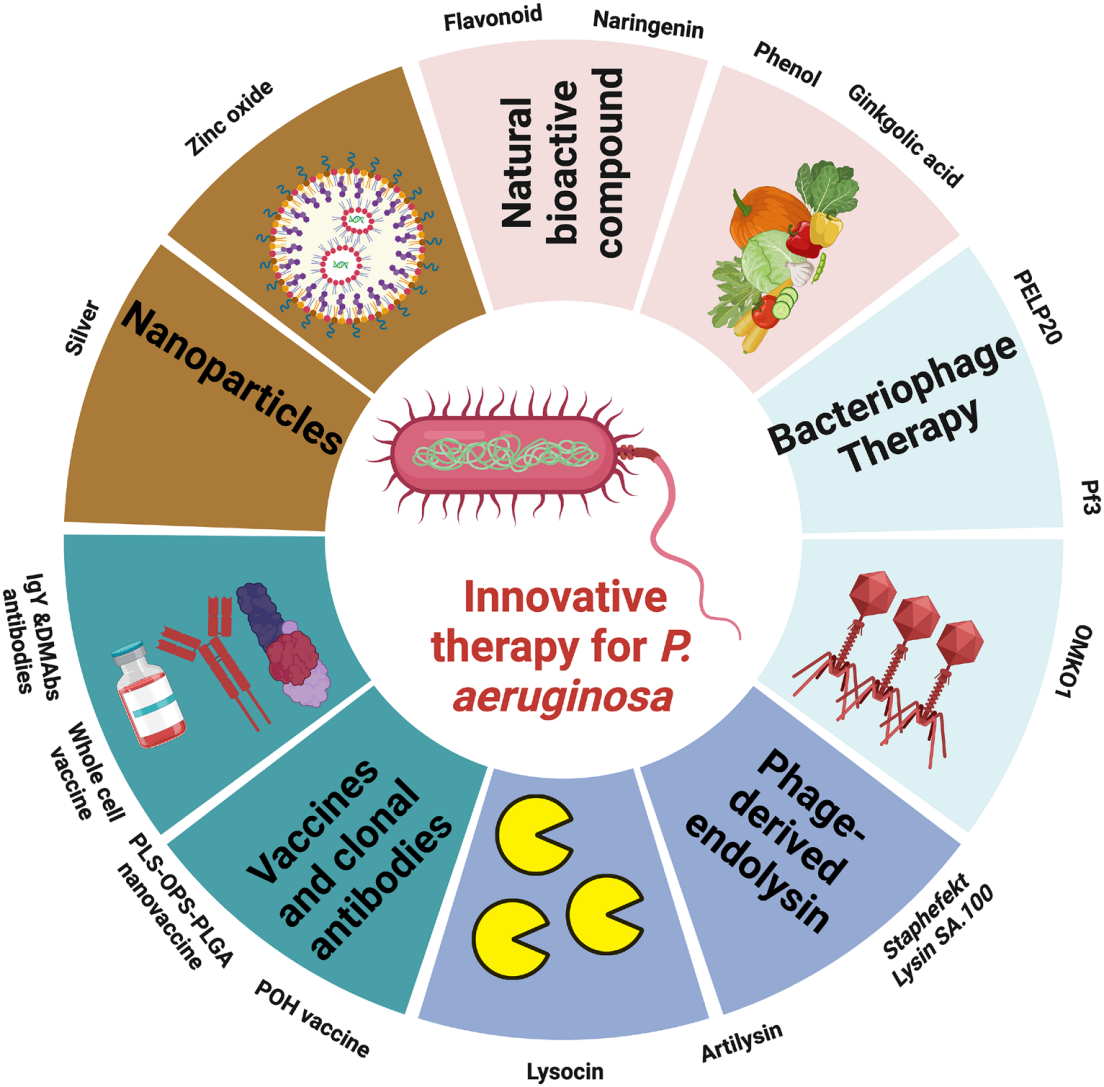
Illustration showing the promising therapies to combat the outbreak of MDR *P. aeruginosa*, the figure was created with biorender.

To combat *P. aeruginosa* infections, healthcare practitioners commonly rely on eight categories of antimicrobial agents. These encompass aminoglycosides (including gentamicin, amikacin, netilmicin, and tobramycin), carbapenems (such as imipenem, meropenem, and doripenem), cephalosporins (notably ceftazidime and cefepime), fluoroquinolones (like ciprofloxacin and levofloxacin), penicillins combined with β-lactamase inhibitors (including ticarcillin-clavulanic acid and piperacillin-tazobactam), monobactams (represented by aztreonam), phosphonic acids (specifically Fosfomycin), and polymyxins (comprising colistin and polymyxin B). Notably, Ceftazidime-avibactam and ceftolozane-tazobactam have received FDA approval and are accessible for clinical use (Bassetti et al., 2019). Additionally, cefiderocol and imipenem-cilastatin/relebactam are presently in the developmental stages (Bassetti and Garau, 2021). Magiorakos et al. established criteria for categorizing *P. aeruginosa* strains based on their resistance profiles. According to these criteria, strains are considered MDR if they exhibit non-susceptibility to at least one agent in three or more antimicrobial categories. Strains are categorized as extensively drug-resistant (XDR) when they display non-susceptibility to at least one agent in all categories except for two or fewer (Magiorakos et al., 2012). Lastly, strains are classified as pandrug-resistant (PDR) if they demonstrate non-susceptibility to all the listed antimicrobial agents (Magiorakos et al., 2012). The excessive and improper use of antibiotics is causing growing concerns in the realm of public health. This misuse not only leads to unnecessary side effects but also contributes to the development of drug-resistant bacteria. Adding to the challenge is the slow and limited progress in developing new antibiotics. As a result, there has been a noteworthy shift in focus over the past decade toward innovative approaches for tackling *P. aeruginosa* infections (Figure 4). These fresh strategies can function independently or in conjunction with traditional treatments, offering a diverse toolbox for combating these infections.

### 5.1 Natural bioactive compounds

Natural Bioactive Compounds (NBCs) are metabolites originating from plants that possess antioxidant, antitumor, anti-inflammatory, and antimicrobial functions (Youns and Abdel Halim Hegazy, 2017; Pai et al., 2022; Elfadadny et al., 2023a). Researchers worldwide are actively investigating novel antimicrobial agents to combat *P. aeruginosa* infections. These studies employ advanced molecular techniques and thorough drug screening to explore the potential of NBCs in countering *P. aeruginosa*. These NBCs have shown promise in inhibiting *P. aeruginosa’s* quorum sensing system, thereby reducing its virulence factors and preventing biofilm formation, a key protective mechanism of *P. aeruginosa*.

Flavonoids, a group of compounds found in plants, play a dual role in this context. They act as antioxidants for the host system, contributing to overall health without toxicity. However, they also act as pro-oxidants when encountered by pathogenic microbes, inducing the formation of reactive oxygen species (ROS) that cause oxidative stress within the bacteria. Researchers have developed an inhibitor called meta-bromo-thiolactone (mBTL) targeting LasR and RhlR, key components of the QS system in *P. aeruginosa* (Eltayb et al., 2022). This inhibitor has demonstrated efficacy both in laboratory settings and *in vivo*. Furthermore, flavonoids have been explored for their inhibitory effects on *P. aeruginosa* biofilm formation, hinting at their potential to disrupt the QS system (Paczkowski et al., 2017). Certain flavonoids with specific structural features, such as dihydroxyl groups in the A ring of flavones, have been identified as inhibitors of LasR and RhlR. Examples include baicalein, naringenin, and quercetin. These flavonoids act allosterically, reducing the binding affinity of LasR and RhlR to regulatory promoters.

Naringenin, a unique QS inhibitor derived from a natural source, is found in plant flavonoids. Its mechanism of action involves reducing the production of QS-regulated virulence factors in *P. aeruginosa* by directly binding to LasR, thereby competing with N-(3-oxo-dodecanoyl)-l-homoserine lactone (HSL), which activates LasR (Hernando-Amado et al., 2020). Consequently, this QS inhibitor can effectively disrupt QS responses only when administered during the early exponential growth phase, allowing naringenin to compete with unbound HSL for binding to LasR. It’s worth mentioning that naringenin is most suitable for targeting *P. aeruginosa* populations at low cellular densities. This scenario may not always align with the clinical infection situation, where bacterial densities tend to be higher. To fully harness the potential of QS inhibition, there is a need for the development of inhibitors capable of targeting *P. aeruginosa* QS signaling irrespective of bacterial density or QS status (Hernando-Amado et al., 2020).

Phenols, specifically 2,4-Di-tert-butylphenol (2,4-DBP), sourced from *Daldinia eschscholtzii*, an endophytic fungus, exhibit significant potential in combating *P. aeruginosa* infections. This compound plays a crucial role in reducing the secretion of pyocyanin and chitinase. Additionally, 2,4-DBP effectively suppresses the expression of genes associated with QS in *P. aeruginosa*, such as *las*I, *las*R, *rhI*I, and *rhI*R (Mishra et al., 2020).

Ginkgolic acid is an NBC found in medicinal plants, which are rich sources of various bioactive compounds known for their antimicrobial, antiviral, antitumor, and other beneficial properties. In a study conducted by Tahrioui et al. (2020), the antivirulent properties of ginkgolic acid were investigated concerning its impact on *P. aeruginosa* in infected *C. elegans* models and found that ginkgolic acid inhibits pyocyanin formation in *P. aeruginosa*. Moreover, ginkgolic acid also influences the mechanical properties of *P. aeruginosa’s* cell membrane by modulating SigX, an extracytoplasmic function σ-factor. SigX plays a role in responding to cell wall stress, contributing to the maintenance of the cell envelope’s integrity, and interacting with specific envelope-related compounds, including ginkgolic acid and hydroginkgolic acid. Exposure of *P. aeruginosa* to ginkgolic acid leads to a significant reduction in the expression of SigX and its associated genes, such as *accA*, *accB*, and *fabY*, which are involved in fatty acid biosynthesis (Tahrioui et al., 2020).

Various other NBCs, like itaconimides, operate on signaling pathways and exhibit the ability to suppress the expression of QS-related genes, thereby mitigating the pathogenicity of *P. aeruginosa*. Additionally, compounds like N-acyl cyclopentamine, the lactone SsoPox enzyme, and N-acyl homoserine lactonase have emerged as potent inhibitors of QS (Chan et al., 2015). Substances such as curcumin and coumarin possess dual capabilities, acting against virulence factors and biofilm formation, while naringenin, gliptin, and taxifolin demonstrate the potential to reduce the expression of QS-related genes (Khayat et al., 2022; Sadaqat et al., 2022). These findings highlight the rich potential of natural compounds in combatting *P. aeruginosa* infections through multifaceted mechanisms.

### 5.2 Bacteriophage therapy

Phages, which are viruses capable of causing bacterial lysis, were initially identified in 1915 by British bacteriologist Frederick Twort. Independently, two years later, Félix d’Herelle in Paris made a similar discovery and introduced the concept of phage therapy. Phage therapy boasts numerous advantages, including its ability to replicate at the infection site, highly specific targeting of bacteria without affecting beneficial flora, minimal side effects compared to other treatments, effectiveness against antibiotic-resistant strains, and ease of administration (Summers, 2012). As an alternative to antibiotics, the use of phages to combat *P. aeruginosa* infections has been extensively researched. Currently, there are 137 distinct phages characterized to target the Pseudomonas genus (Pires et al., 2015). Numerous *in vitro* and *in vivo* studies have explored the efficacy of phages against chronic *P. aeruginosa* infections. Phage therapy offers a notable advantage over antibiotic treatment due to its effectiveness against *P. aeruginosa* biofilms. A recent study demonstrated that phage PELP20 effectively eradicated *P. aeruginosa* strain LESB65, isolated from *CF* patients, within an artificial sputum medium biofilm model *in vitro*. It also significantly enhanced bacterial clearance in a mouse model of chronic lung infection caused by *P. aeruginosa* (Waters et al., 2017). Significantly, pretreating hydrogel-coated catheters with phage M4 substantially reduced the formation of *P. aeruginosa* biofilms. Another advantage of phage therapy lies in the potential to genetically engineer phages as delivery vehicles for antimicrobial agents, thereby enhancing treatment efficacy (Fu et al., 2010). Over the past decade, researchers have tested genetically engineered *P. aeruginosa* phages. For instance, the *P. aeruginosa* phage Pf3 was genetically modified into a nonlytic, nonreplicating phage, Pf3R, by replacing an export protein gene with a BglII restriction endonuclease gene. Pf3R efficiently killed *P. aeruginosa* PAO1 *in vitro*, similarly to the lytic phage Pf3, while significantly reducing endotoxin release. Moreover, Pf3R-treated mice displayed an increased survival rate compared to those treated with Pf3, likely due to reduced endotoxin levels and fewer host inflammatory responses (Hagens et al., 2004). An alternative strategy in phage therapy involves using phages to influence the evolution of antibiotic resistance, favoring the development of phage resistance while simultaneously restoring antibiotic susceptibility. As an example, the lytic Myoviridae bacteriophage, OMKO1, employs the bacterial multidrug efflux systems MexAB and MexXY, specifically OprM, as its receptor-binding site. Selecting for resistance to OMKO1 phage attack triggers an evolutionary trade-off in MDR *P. aeruginosa*. This trade-off results in a modification of the efflux pump mechanism, which in turn increases the sensitivity of the bacteria to various antibiotics, including ciprofloxacin, tetracycline, ceftazidime, and erythromycin—four drugs representing different antibiotic classes (Chan et al., 2016). Phage steering becomes achievable when the binding receptor for the bacteriophage is implicated in both antibiotic resistance and phage resistance. This approach offers the advantage of employing two distinct and opposing mechanisms to combat bacterial infections (Gurney et al., 2020).

Despite the proven effectiveness of phages against bacterial infections *in vitro* and animal models, the number of clinical trials for phage therapy remains limited. This is primarily attributed to concerns about phage clearance after treatment, impurities in phage preparations, poor stability of phage preparations, and a lack of detailed knowledge about phage mechanisms of action and the potential for bacterial resistance to phages (Jaglan et al., 2022). Clinical trials examining the use of phages against *P. aeruginosa* infections in patients with venous leg ulcers, burn wounds, and otitis have reported no adverse effects.

### 5.3 Phage-derived endolysin

Among the recent alternative antibacterial agents being studied now are phage lytic antimicrobial peptides (AMPs). These are bacterial peptidoglycan-degrading enzymes produced by bacteriophages and cause instantaneous cell death (Briers, 2019). Endolysins are lytic enzymes produced during the late phase of the lytic bacteriophage replication cycle to target the bacterial cell walls for progeny release. The use of endolysins as antimicrobial agents against *P. aeruginosa* presents a promising avenue for combatting this resilient bacterium (Furusawa et al., 2016). However, deploying endolysin therapy against *P. aeruginosa* is not straightforward, primarily due to the protective outer membrane that shields the bacterial cell from lysin attack. The outer membrane of Gram-negative bacteria, including *P. aeruginosa*, consists of phospholipids and lipopolysaccharides. LPS molecules are held together by phosphate bonds, making them challenging to breach. To enable endolysins to access the peptidoglycan cell wall and effectively target *P. aeruginosa*, researchers have employed creative strategies. One such method involves the use of ethylenediaminetetraacetic acid (EDTA) to permeabilize the Gram-negative cell membrane (Murray et al., 2021). EDTA is a chelating agent that removes divalent cations, essential for stabilizing the outer membrane, from their binding sites. This disrupts the membrane’s integrity, rendering it vulnerable to endolysin attack. Permeabilizing the outer membrane can also be achieved through mechanical means, such as high hydrostatic pressure (HPP). HPP has been employed to sensitize Gram-negative bacteria, including *P. aeruginosa*, to molecules like bacteriocins and antimicrobial peptides by optimizing pressure and time parameters (Prudêncio et al., 2015). Another approach involves fusing endolysins with components such as antimicrobial peptides that facilitate penetration of the peptidoglycan layer. This fusion strategy enhances the endolysin’s ability to reach its target within the bacterial cell. In the case of *P. aeruginosa*, a bacterium renowned for its antibiotic resistance, endolysin therapy has shown promise. Researchers isolated a phage-derived endolysin called LysPA26 in 2017, which exhibited potent lytic activity against MDR strains of *P. aeruginosa*. Remarkably, this endolysin achieved a reduction in bacterial numbers of up to four log units in just 30 min. Furthermore, LysPA26 demonstrated stability under varying pH and temperature conditions (Guo et al., 2017). Notably, this endolysin exhibited a broad spectrum of activity, effectively targeting other Gram-negative bacteria such as *Klebsiella pneumoniae*, *Acinetobacter baumannii*, and *Escherichia coli*. The success of LysPA26 underscores the potential of endolysin therapy in addressing MDR *P. aeruginosa* infections. Its selective targeting of Gram-negative bacteria without the use of antibiotics holds promise as a primary therapeutic option for the future. Currently, not enough data is available regarding the safety profile of endolysins. Also, there is a lack of information about the pharmacokinetics and pharmacodynamics of endolysins as possible medications. Cellular toxicity and cell-penetrating properties of phage-derived endolysins should be studied before being administered as human therapeutic medications (Nandi et al., 2022). *In vivo* studies are limited to topical models with some concerns about systemic administration related to their short half-life, immunogenicity, and release of proinflammatory components during bacteriolysis which should addressed before further development (Lai et al., 2020).

### 5.4 Vaccine development

Developing effective vaccines against *P. aeruginosa* infections is a crucial strategy to mitigate their consequences and reduce the excessive use of antibiotics, which can lead to antibiotic resistance. Various types of vaccines are under development to enhance the immune response against different elements involved in the infection process. These vaccines often target components of the bacterial surface, such as Opr, and various polysaccharides, including LPS, mucoid exopolysaccharide, and O-polysaccharides (Sharma et al., 2011). Additionally, they focus on structures critical for *P. aeruginosa* ‘s adhesion and movement, such as flagella and pili, as well as several virulence factors like TTSS, exotoxin A, or proteases. Developing effective vaccines for *P. aeruginosa* is challenging due to the substantial variability among Pseudomonas species, the complexity of the infection process, and the intricate interaction between the pathogen and the host immune response (Killough et al., 2022). During phases I, II, and III studies, certain vaccine candidates have proven insufficient in providing broad coverage against different *P. aeruginosa* strains or have exhibited low immunogenicity or safety concerns. Currently, research is ongoing to develop novel *P. aeruginosa* vaccines. For example, a novel *P. aeruginosa* vaccine called PcrV28-294-OprI25-83-Hcp11-162 (POH) has been evaluated for its protective efficacy. This vaccine contains components like PcrV, OprI, and a vital part of the type VI secretion system, Hcp1 (Yang et al., 2017). Studies showed that POH vaccination significantly triggered T-cell responses, proliferation, and protected mice against clinical *P. aeruginosa* strains. The development of multivalent vaccines is also being explored as a potential means of protecting against *P. aeruginosa* infections in the future. Furthermore, efforts have been directed toward vaccines that target notorious virulence factors responsible for evading the host immune response. Different formulations of LPS extracts have been experimented in clinical trials since 1970 including Pseudogen^®^ and although showing some success in reducing mortality and sepsis in burn patients, it did not show clinical efficacy and did not show any reduction in colonization in *CF* patients (Langford and Hiller, 1984). Aerugen^®^ was another successful candidate consisting of a component of LPS, the O-polysaccharide from eight *P. aeruginosa* serotypes and exotoxin A and showed favorable outcomes in *CF* patients (Lang et al., 2004). One promising approach involves using LPS and Oligopolysaccharides (OPS) antigens conjugated with Poly Lactic-co-Glycolic Acid (PLGA) nanoparticles as nano-vaccines. These nano-vaccines have shown the potential to stimulate both cellular and humoral immune responses against *P. aeruginosa* infections (Wusiman et al., 2022). Additionally, nano-vaccines containing antigens from bacterial lysates of *P. aeruginosa* and membrane antigens from double-layered membrane vesicles have demonstrated efficiency in preventing infections by drug-resistant *P. aeruginosa* strains. Alginate, a significant contributor to *P. aeruginosa* pathogenesis, is also being explored as a target for therapeutic vaccines. Mannuronic acid tetra saccharide, an antigen epitope, has been used to induce immunity against *P. aeruginosa* in mice. Hybrid proteins composed of the full-length V-antigen (PcrV) and the C-terminal domain of exoenzyme S (ExoS) from *P. aeruginosa*, along with adjuvants like alum and monophosphoryl Lipid A, have been considered as vaccine candidates to protect against urinary tract infections caused by *P. aeruginosa* (Asadi Karam et al., 2022). Whole-cell vaccines inactivated by X-ray irradiation, containing nucleic acids and 8-hydroxyguanosine, have demonstrated humoral and innate immune responses, leading to reduced infection levels in mouse models (Ma et al., 2021). Moreover, the concept of multi-target antigens has been explored. Triple-target antigens have been designed to stimulate protective and neutralizing antibodies against multiple pathogens, including *P. aeruginosa* (Inoue et al., 2023). These antigens combine epitopes from different bacterial species and have the potential to provide broad-spectrum protection. Vaccines represent a promising strategy for preventing *P. aeruginosa* infections and reducing their impact. They can induce protective responses through active or passive immunization and, in some cases, may even cross-react to protect against related pathogens. Although no effective vaccine is currently available for clinical use despite studies being performed over the last 50 years, the development of glycoconjugate vaccines, protein engineering techniques, and innovative carrier proteins has opened new avenues for creating effective vaccines against *P. aeruginosa*. Progress has been achieved in pre-clinical studies including antigen discovery, adjuvant use, and novel delivery systems, however, the wealth of virulence factors, its large genome size and great adaptability, and its changing phenotypes across acute and chronic infections all form challenges for vaccine development (Killough et al., 2022).

### 5.5 Monoclonal antibodies production

Monoclonal antibodies (mAbs) have emerged as a valuable strategy in combatting *P. aeruginosa* infections, particularly for high-risk individuals who may not benefit from vaccines or traditional antibiotics (Kang et al., 2023). These mAbs provide an alternative treatment avenue by directly targeting *P. aeruginosa* and offering immediate protection. They can be especially effective when used alongside prophylactic vaccines. One notable application of mAbs is their ability to tackle antimicrobial resistance within biofilms, where bacteria reside. These antibodies have been developed to disrupt protective biofilms, making *P. aeruginosa* and its co-pathogens vulnerable to subsequent antibiotic treatment. Additionally, mAbs targeting specific epitopes like DNABII proteins or type IV pilus have shown promise in impairing biofilms formed by *P. aeruginosa* and related pathogens (Sanya et al., 2023). Chicken egg yolk immunoglobulins, known as IgY antibodies, have garnered special attention for passive immunization due to their unique properties. IgY antibodies do not cross-react with mammalian IgG or activate the complement system. They offer high antigen-specific production yields without causing disease resistance and can be easily administered to humans. For example, IgY antibodies raised against the T3SS translocating protein PcrV from *P. aeruginosa* have demonstrated enhanced bacterial killing and reduced invasion in murine models of acute pneumonia and burn wounds (Ranjbar et al., 2019). Moreover, a synergistic effect has been observed when combining anti-*P. aeruginosa* IgY with beta-lactam antibiotics, suggesting a potential combination therapy for MDR *P. aeruginosa* infections. In severe cases of *P. aeruginosa* pneumonia, mAbs can be coupled with antibiotics to enhance therapeutic efficacy (Sanches et al., 2022). DNA-delivered monoclonal antibodies (DMAbs) produced *in vivo* have been effective in protecting mice from lethal pneumonia caused by aggressive *P. aeruginosa* strains (Patel et al., 2017). These antibodies not only reduced bacterial colonization but also acted synergistically with commonly used antibiotics, such as meropenem. Importantly, they exhibited stability and hold promise for treating high-risk patients, including those with chronic diseases and infections refractory to broad-spectrum antibiotics. However, it’s crucial to acknowledge that therapeutic monoclonal antibodies have their limitations. For instance, some mAbs, like MEDI3902, have not proven effective in mitigating *P. aeruginosa* nosocomial pneumonia in clinical trials (Chastre et al., 2022). Additionally, immunogenicity and protective efficacy can vary based on the antibody type and dosing. For example, IgY antibodies may protect certain infection models but not others, depending on the dose and specific target. Current research efforts are focused on using mAbs to prevent infections in high-risk patients and modulate virulence rather than solely aiming for bacterial clearance. Several mAbs are in various stages of development and clinical trials, targeting different aspects of *P. aeruginosa* infections, such as protein secretion systems and exopolysaccharides. These mAbs hold promise as adjunctive strategies in managing *P. aeruginosa* infections, particularly in critical care settings.

### 5.6 Nanoparticles

Nanoparticles have emerged as a promising frontier in the battle against *P. aeruginosa*. These minuscule materials, typically measuring less than 100 nanometers, have attracted significant attention for their potential in treating *P. aeruginosa* infections. Their diminutive size and high surface area-to-mass ratio make them ideal candidates for a wide range of chemical, biological, and biomedical applications (Al Saqr et al., 2021; Varma et al., 2023). One key advantage of nanoparticles is their ability to penetrate the protective barriers of bacterial cells. *P. aeruginosa* possesses an outer membrane rich in LPS, creating a formidable defense. However, researchers have developed methods to breach this barrier, allowing nanoparticles to access the bacterial cell wall. One technique involves using EDTA, a chelating agent that removes divalent cations stabilizing the outer membrane. This disruption leaves the membrane vulnerable to nanoparticle attack, allowing them to effectively target and combat *P. aeruginosa*. Another approach involves engineering nanoparticles to carry antimicrobial agents directly to their target. Porous silicon nanoparticles, for instance, have been designed to transport novel antimicrobial peptides fused with synthetic bacterial toxins. These engineered nanoparticles have demonstrated the ability to enhance survival rates and bacterial clearance in mouse models of *P. aeruginosa* lung infections (Patel and Hunt, 2023). Moreover, nanoparticles can serve as carriers for antibiotics, significantly augmenting their efficacy. Silver nanoparticles, when coupled with antibiotics like ampicillin, exhibit enhanced killing rates of ampicillin-resistant *P. aeruginosa* isolates *in vitro*. Silver nanoparticles, in particular, have shown remarkable promise as antimicrobial agents against *P. aeruginosa*. They release silver ions, which disrupt crucial bacterial enzymatic systems, including DNA synthesis, leading to bacterial cell death (Palau et al., 2023). Notably, these silver nanoparticles have demonstrated significant antimicrobial effects on clinical strains of *P. aeruginosa*, effectively eradicating the bacterium and inhibiting its growth in laboratory settings. Furthermore, they exhibit low cytotoxicity toward mammalian cells, a crucial consideration for potential clinical applications. Additionally, nanoparticles have been used as antigen delivery systems as a cost-effective approach to vaccine development. However, it’s important to recognize that the use of nanoparticles is not without challenges. Their reactivity, stemming from their high surface area, can lead to unintended reactions within the human body. Nanoparticles can also migrate to distant organs, potentially causing systemic toxicity. Inhalation of nanoparticles has been associated with pulmonary inflammation and potential cardiovascular effects, making their use in treating bacterial pulmonary infections a matter of careful consideration (Missaoui et al., 2018). To advance the field, future research should focus on material selection, nanoparticle size, and administered doses to optimize their therapeutic efficacy while minimizing potential risks in the clinical aspect.

## 6 Conclusion and future directions

Mitigating the resistance of *Pseudomonas aeruginosa* is a formidable challenge, prompting the exploration of innovative therapeutic avenues. Future directions in research and treatment focus on advanced adjuvants and combination therapy. Although innovative therapies exhibit advantages over current antibiotics, some limitations still persist. Notably, we view endolysins or enzybiotics as superior, owing to their efficacy in combatting bacterial resistance. The challenge of penetrating *P. aeruginosa’s* outer membrane is addressed through proposed solutions. Fusion with antimicrobial peptides enhances endolysins’ effectiveness, targeting the bacterium’s cell wall and inner membrane. Administering endolysins with permeabilizer agents facilitates membrane passage, optimizing antimicrobial effects. Precision medicine emerges as a promising future direction, leveraging genomic profiling for tailored treatments. Real-time antibiograms aid clinicians in data-driven decisions based on strain-specific resistance profiles. This personalized approach optimizes treatment outcomes and restricts unnecessary antibiotic use. The potential of phage therapy, biotechnology, and synthetic antimicrobial peptides in combating *P. aeruginosa* infections is underscored. Bacteriophages offer targeted solutions, with ongoing research into identifying and engineering phages with broad-spectrum activity. Biotechnological advancements enable synthetic peptides to disrupt bacterial resistance effectively.

In conclusion, managing *P. aeruginosa* infections remains a complex challenge. Its adaptability and antibiotic resistance mechanisms, resulting in multidrug-resistant strains, hinder conventional treatments. Biofilm formation and persistent cells contribute to persistent infections, especially in conditions like cystic fibrosis. Advances in antibiotics and drug delivery methods face continual challenges from *P. aeruginosa* ability to develop novel resistance mechanisms. The global misuse of antibiotics intensifies the need for alternative therapies. Non-antibiotic strategies, including quorum sensing inhibition, phage therapy, and nanoparticles, show promise but face translation hurdles. The multifaceted battle requires combining innovative strategies with traditional antibiotics. Combination therapies, especially for immunocompromised patients, hold potential.

## Author contributions

AE: Conceptualization, Supervision, Investigation, Resources, Visualization, Writing – original draft, Writing – review & editing. RR: Data curation, Resources, Writing – original draft, Writing – review & editing. MaA: Writing – review & editing, Supervision, Investigation, Resources, Funding acquisition. FB: Formal analysis, Investigation, Writing – original draft, Writing – review & editing. EI-A: Data curation, Investigation, Resources, Writing – original draft, Writing – review & editing. AF: Investigation, Resources, Writing – original draft, Writing – review & editing. AH: Data curation, Investigation, Writing – original draft, Writing – review & editing. PR-E: Investigation, Resources, Supervision, Writing – original draft, Writing – review & editing. MoA: Data curation, Investigation, Writing – original draft, Writing – review & editing. SAZ: Writing – review & editing, Supervision, Investigation, Resources, Funding acquisition. WN: Conceptualization, Investigation, Visualization, Resources, Writing – original draft, Writing – review & editing.
